# No significant clinical advantage of robot-assisted versus laparoscopic fundoplication was observed in this single-centre retrospective cohort

**DOI:** 10.3389/fsurg.2026.1970501

**Published:** 2026-10-26

**Authors:** Mike Ralf Langenbach, Ehab Ibrahim, Mustafa Eltohamy, Lars Daenenfaust

**Affiliations:** 1 Evangelisches Krankenhaus Lippstadt GmbH, Lippstadt, Germany; 2 Universitat Witten/Herdecke, Witten, Germany; 3 Universitat zu Koln, Cologne, Germany

**Keywords:** fundoplication, hiatal hernia, laparoscopic methods, reflux, robotic surgery

## Abstract

**Background:**

Robot-assisted laparoscopic fundoplication (RALF) has been proposed as a promising alternative to conventional laparoscopic fundoplication (CLF). Because comparative data are still sparse, we compared RALF and CLF in a cohort study. At our centre, RALF was introduced in late 2022.

**Methods:**

RALF was performed on a DaVinci robot. Clinical data were abstracted from clinical records of all patients who underwent surgery for gastroesophageal reflux disease (GERD) between January 2020 and June 2025. Statistical analysis was used to compare RALF and CLF and to analyse the learning curve of RALF over time.

**Results:**

More patients in the RALF group (total *n*=107) than in the CLF group (*n*=126), had an upside-down stomach (55% vs. 19%) and hiatal defect sizes were larger. In 6 RALF patients, the procedure had to be converted or abandoned. Operating room (OR) time was longer with RALF (mean 251 min) than with CLF (177 min, p 0.001). Adverse events during surgery and during hospitalization were similar between the groups, but hospital stay was slightly longer in RALF than in CLF patients (median 7 vs. 6 days, *p*=0.12). Readmissions for recurrent symptoms (20% vs. 20%) or incisional hernia (10% vs. 14%) were similar between RALF and CLF. An uneventful clinical course (i.e., “textbook outcome”) was seen in 57 (53%) RALF and 66 (52%) CLF patients. In logistic regression, only anaesthesiologic risk was associated with a non-textbook outcome. A learning curve for RALF was not found, as mean OR time was longest in the second half of 2024 and the first half of 2025 (*p*=0.005).

**Conclusions:**

For patients requiring anti-reflux surgery, RALF prolongs surgery times but fails to offer clear advantages over CLF. As in the present study RALF was applied in more complicated cases and still gave grossly similar results as CLF, the possibility remains that RALF is a minor improvement as compared to CLF.

## Introduction

Gastroesophageal reflux disease (GERD) is common and often requires surgical treatment, if pharmacological therapy fails to alleviate the symptoms (1, 2). Since the beginning of this century, laparoscopic fundoplication is universally accepted as a standard intervention for treating GERD (3). For hiatal hernia, which are often found in patients with GERD, laparoscopic repair is similarly well accepted and effective.

In parallel, robotic surgery has become commonplace in many operating rooms. Nevertheless, despite its popularity in other fields, robot-assisted anti-reflux surgery is still a controversial issue. The lack of large-scale randomized clinical trials (RCTs) coupled with the heterogeneity of surgical techniques and patient populations, contributed to the slow evolution of robot-assisted procedures in the treatment of GERD. This is particularly true for robot-assisted laparoscopic fundoplication (RALF). According to the most recent meta-analyses (4–6) the scientific evidence on this topic consists of five RCTs with sample sizes between 20 and 50 patients (7–11) and about 30 non-randomized comparative studies with a total sample size of about 5000 patients. Because the RCTs are small in size and the majority of the non-randomized studies failed to adjust for baseline differences, the current knowledge regarding laparoscopic anti-reflux surgery is limited in quantity and validity. From the available data, however, it does appear that RALF does not confer any convincing clinical advantage. The clearest finding was that robotic surgery requires more time in the operating room and leads to higher costs. Recently published studies confirmed these results (12–15).

The aim of the present study therefore was to compare robot-assisted anti-reflux surgery (including RALF) with conventional laparoscopic anti-reflux surgery in patients with symptomatic GERD. By applying multivariate statistics, we strived for valid comparisons of short- and medium-term outcomes, and we also aimed at examining the influence of the surgical learning curve on the results.

## Methods

### Study design

In this retrospective single-centre cohort study, robot-assisted and conventional laparoscopic anti-reflux surgery were compared. Due to the retrospective observational nature of the study and the pseudonymized data collection, neither an ethics committee approval nor informed consent from the patients was required. For transparent reporting, we followed the STROCCS (Strengthening the Reporting Of Cohort, cross-sectional, and Case-control studies in Surgery) guideline (16).

The sample size resulted from our decision to limit the analysis to a 5-year observation period. It was expected that, after starting with RALF in late 2022, about 3 years were sufficient for both, overcoming the learning curve of robot-assisted surgery and accumulating enough patient data for meaningful analysis. Based on the hospital's electronic health records (EHR), a consecutive sample of all patients undergoing anti-reflux surgery between January 2020 and June 2025 was selected. All patients’ data were also entered prospectively in the HerniaMed registry (17).

### Patients

Patients were eligible for the study if they had been admitted and were scheduled for elective anti-reflux surgery. Adult patients of both sexes and all ages were included consecutively. All study participants suffered from GERD symptoms and had tried conservative therapy before accepting surgery. Surgical techniques were selected based on the preferences of surgeons and the individual characteristics of each patient. Robotic assistance was generally preferred for more complex cases, such as recurrences and large hiatal hernias.

Preoperative work-up, anaesthesia, and postoperative care were essentially the same in both groups. Preoperative diagnostic tests changed with time, but without specific difference between patients who were planned for robot-assisted or laparoscopic surgery. All patients were contacted for 1 month, 6 months, and 12 months visits. The 12 months data are not yet available for the 30 patients operated on in 2025, but otherwise, completeness of follow-up was over 95%.

### Procedures

The surgeons who performed the procedures were well-qualified in laparoscopic surgery. Before using the robot, every surgeon completed the full training programme that is recommended by the manufacturer (Intuitive Surgical, Sunnyvale CA), including training on animals and on-site feedback by a very experienced surgeon. None of the surgeons, however, had previous experience with robotic assistance in any other surgical procedure.

In the conventional laparoscopic group, total (i.e., Nissen) or posterior partial (i.e., Toupet) fundoplication were performed, with Toupet fundoplication being generally preferred. Gastropexy, i.e., fundophrenopexy, was added if deemed necessary by the surgeon. Concomitant hiatal hernias were repaired by applying a mesh-reinforced, tension-free technique. In 2024, we stopped mesh reinforcement and relied on crural suturing alone (18).

In the robot-assisted group, a DaVinci robot Xi® (Intuitive Surgical, Sunnyvale CA) was used. The principles for selecting surgical techniques were the same in robot-assisted and conventional laparoscopic surgery. For example, tacker fixation was replaced by fibrin glue fixation during the course of the study, but this change in technique occurred at the same time in both groups.

### Outcomes

Demographics and clinical baseline characteristics were collected without already being aware of the patient's clinical course. All data were recorded by a single person. Preoperative diagnostic examinations were counted, if being done within the last 4 weeks before surgery. The hiatal defect area was calculated by using the standard elliptic formula.

According to the retrospective explorative character for the study, no primary outcome was defined. Intraoperatively, any deviation from the normal surgical procedure was recorded as a complication, if this deviation could have led to a patient-relevant serious adverse event if appropriate action had not been taken or circumstances had been less fortunate. In the postoperative period, any event was counted as a complication, if this event reached grade II or higher on the Clavien-Dindo classification.

As a textbook outcome (i.e., an uneventful and successful treatment), we defined a postoperative course fulfilling four criteria: i) no complication within hospital stay, ii) hospital discharge in less than 10 days, iii) no readmission for symptom recurrence or hernia repair during follow-up, and iv) no death. Intraoperative adverse events (e.g., bleeding) and changes in technique (e.g., conversion) did not necessarily prevent a textbook outcome, as only patient-relevant events were counted.

### Statistical analysis

Data were collected in a spreadsheet computer file and analysed in a statistical software (PSPP V2.0). Demographic and outcome variables were compared between the two groups by applying either Student's test for continuous or Fisher's exact test for categorical variables. The chi-square test was applied to compare among more than 2 groups. If a non-Normal distribution of a continuous variable was visible, the Mann–Whitney test was applied and medians (with ranges) rather than means (with standard deviations) were reported. On an exploratory basis, *p* values of < 0.05 were considered significant.

Reaching a textbook outcome was used as an overarching indicator in multivariate analysis. Logistic regression modelling was applied to examine the role of confounding variables on this outcome. This analysis aimed at reducing the influence of baseline imbalances and obtaining a more valid estimate of the true effectiveness of RALF vs. CLF. As potentially relevant confounders, we selected age, gender, ASA (American Society of Anesthesiologists) score, body mass index (BMI), recurrency status, and hiatal defect size. Presence of an upside-down stomach was not taken as a confounder, because of its very close association with hiatal defect size. For the ease of results interpretation, all variables were dichotomized. The cut-off values for dichotomization were chosen without knowledge of the results.

## Results

There were 107 patients in the RALF group and 126 in the CLF group. Robot assistance became more common over time (*p* < 0.001, Table 1). It was applied slightly more often in patients with recurrent GERD. Patients in the RALF group had a shorter symptom duration, but larger hiatal defect sizes (Table 2). With regard to preoperative imaging, computed tomography (CT) scanning was done more often in the RALF group, whereas the barium swallow was preferred in the CLF group. This difference was largely explained by a time trend (Table 3). Application of both diagnostic tests was twice as likely among patients with an upside-down stomach (23 of 83, 28%) than in those without (21 of 150, 14%, *p* = 0.014).

**Table 1:** Baseline demographic characteristics .

	Laparoscopic	Robot-assisted	*p* value
No. of patients	126	107	-
Year of surgery			<0.001
2020 and 2021	33 (26%)	0	
2022	44 (33%)	2 (2%)	
2023	30 (24%)	33 (31%)	
2024	17 (14%)	46 (43%)	
2025	4 (3%)	26 (24%)	
Females	76 (60%)	75 (70%)	0.13
Age (mea*n* ± SD)	59.9 ± 14.9	63.1 ± 11.7	0.07
BMI (mean ± SD)	28.3 ± 5.0 (range 19.5 to 43.6)	28.8 ± 4.8 (range 19.1 to 41.0)	0.46
ASA classification			0.43
1	0	1 (<1%)	
2	91 (72%)	71 (66%)	
3	35 (28%)	34 (32%)	
4	0	1 (<1%)	
Comorbidities			
Coronary heart disease	14 (11%)	9 (8%)	0.52
Chronic heart failure	3 (2%)	8 (8%)	0.12
Obstructive lung disease	7 (6%)	4 (4)	0.56
Anticoagulation	11 (9%)	13 (12%)	0.40
Renal insufficiency	2 (2%)	3 (3%)	>0.99
Prior abdominal surgery			
Laparoscopic	42 of 125 (34%)	51 of 105 (49%)	0.023
Open	51 of 125 (41%)	56 of 105 (53%)	0.064
Neither	54 of 125 (43%)	32 of 105 (31%)	0.056

**Table 2:** Preoperative disease severity and intraoperative findings.

	Laparoscopic (*n* = 126)	Robot-assisted (*n* = 107)	*p* value
Reflux symptoms	110 (87%)	99 (93%)	0.20
Proton pump inhibitors (PPI) medication	108 (86%)	98 (92%)	0.22
Preoperative symptom duration (median, months)	36 (IQR 10 to 72; range 1 to 480)	10 (IQR 6 to 36; range 1 to 600)	<0.001
In subgroup of recurrent cases	12 (IQR 8 to 78; range 2 to 168; *n* = 21)	6 (IQR 4 to 12; range 2 to 600); *n* = 29)	0.017
In subgroup of new cases	36 (IQR 12 to 72; range 1 to 480); *n* = 105	12 (IQR 6 to 48; range 1 to 300; *n* = 78)	0.003
Recurrent disease (following previous surgery)	21 (17%)	29 (27%)	0.06
Computed tomography (CT) done preoperatively	41 (33%)	77 (72%)	<0.001
In subgroup of recurrent cases	10 of 21 (48%)	24 of 29 (83%)	0.014
In subgroup of new cases	31 of 105 (31%)	53 of 78 (68%)	<0.001
Barium swallow done preoperatively	100 (79%)	52 (49%)	<0.001
In subgroup of recurrent cases	13 of 21 (62%)	11 of 29 (38%)	0.15
In subgroup of new cases	87 of 105 (83%)	53 of 78 (68%)	<0.001
Upside-down stomach	24 (19%)	59 (55%)	<0.001
In subgroup of recurrent cases	2 of 21 (10%)	13 of 29 (45%)	0.011
In subgroup of new cases	22 of 105 (21%)	41 of 78 (53%)	<0.001
Hiatal defect size			
Length (in cm)	5.1 ± 2.4	8.8 ± 4.1	<0.001
Width (in cm)	3.5 ± 1.6	5.7 ± 2.4	<0.001
Area (in cm^2^)	16.2 ± 15.3	44.9 ± 32.6	<0.001

**Table 3:** Change in preoperative diagnostics over time

	2020 (*n* = 12)	2021 (*n* = 21)	2022 (*n* = 44)	2023 (*n* = 63)	2024 (*n* = 63)	2025 (*n* = 30)	*p* value
Barium swallow	10 (83%)	16 (76%)	34 (77%)	37 (59%)	33 (52%)	22 (73%)	0.044
CT scan	5 (42%)	9 (43%)	14 (32%)	33 (52%)	39 (62%)	18 (60%)	0.007
Both diagnostics	3 (25%)	4 (19%)	7 (16%)	9 (14%)	12 (19%)	10 (33%)	0.47
Neither of both	0	0	3 (7%)	2 (3%)	3 (5%)	0	-

All 6 conversions occurred in the RALF group. All these patients had had previous abdominal surgery. One case had to be converted, because a tumour was found intraoperatively, which later turned out to be a lymphoma. The other 5 conversions were due to intraoperative difficulties. More than 80% in both groups, however, underwent surgery without any intraoperative complication (Table 3). The duration of surgery was 74 min longer in the RALF than in the CLF group. All phases in the operating room (i.e., set up, surgery, post-operative care) took longer when robot assistance was used.

After surgery, more patients of the RALF group as opposed to the CLF group were admitted to the intensive care unit for observation (Table 4). This was caused by our postoperative protocol for patients with comorbidities und more complex cases as we found in the RALF group. The incidence of complications and the length of hospital stay were nevertheless similar between the two groups (Table 5). After discharge, a relatively high proportion of patients had to be readmitted for recurrent symptoms or incisional hernia repair, but again without any statistical difference between the groups.

**Table 4:** Intraoperative and postoperative results.

	Laparoscopic (*n* = 126)	Robot-assisted (*n* = 107)	*p* value
Mesh implanted	57 (45%)	59 (55%)	0.15
Ventral mesh	24 (19%)	40 (37%)	0.002
Dorsal mesh	54 (43%)	59 (55%)	0.07
Tacker fixation	31 (25%)	1 (1%)	<0.001
Fibrin glue fixation	27 (21%)	58 (54%)	<0.001
Surgical techniques			
Dor fundoplication	0	5 (5%)	0.02
Toupet fundoplication	70 (56%)	70 (65%)	0.14
Nissen fundoplication	59 (39%)	12 (11%)	<0.001
Gastropexy	9 (7%)	14 (13%)	0.19
Fundophrenicopexy	60 (48%)	69 (65%)	0.012
Surgery completed as planned			0.009
Yes	126 (100%)	101 (94%)	
No, i.e., conversion necessary	0	6 (6%) 5 to open, 1 to lap	
Intraoperative complications			
None	105 (83%)	87 (81%)	0.73
Any organ lesion	16 (13%) 5 gastroesophageal, 2 lung, 9 pleura, 2 trachea, 1 vagus, 0 heart	19 (18%) 10 gastroesophageal, 1 lung, 7 pleura, 0 trachea, 3 vagus, 2 heart	0.36
Major bleeding	5 (4%)	2 (2%)	0.46
Total duration of surgery (in minutes)	177 ± 35 (range 94 to 279)	251 ± 64 (range 125 to 460)	<0.001
Duration of set up	30 ± 10	45 ± 24	<0.001
Duration of procedure	133 ± 33	187 ± 61	<0.001
Duration post-procedure	14 ± 6	21 ± 8	<0.001

**Table 5:** Postoperative results.

	Laparoscopic (*n* = 126)	Robot-assisted (*n* = 107)	*p* value
ICU admission after surgery	18 (14%)	35 (33%)	0.001
Length of hospital stay (median, days)	6 (IQR 5 to 8; range 4 to 30)	7 (IQR 6 to 8; range 5 to 52)	0.12 (n-p)
Barium enema after surgery			
Normal	110 (87%)	85 (79%)	0.11
Stenosis	10 (8%)	11 (10%)	0.65
Perforation	1 (1%)	1 (1%)	> 0.99
Not done	5 (4%)	10 (9%)	0.11
Postop adverse events			
Dysphagia	20 (16%)	14 (13%)	0.58
Reflux	29 (23%)	25 (23%)	>0.99
Gastroparesis	6 (5%)	8 (8%)	0.67
Surgical site infection	1 (1%)	2 (2%)	0.60
Incisional hernia	4 (5%)	6 (6%)	0.76
Any additional surgery	23 (18%)	19 (18%)	>0.99
Death	1 (1%)	1 (1%)	>0.99
Readmission after discharge			
For recurrent symptoms	25 (20%)	21 (20%)	>0.99
For incisional hernia repair	18 (14%)	11 (10%)	0.43
Textbook outcome	66 (52%)	57 (53%)	0.90

The comparison of half-year intervals failed to show any improvement over time (Table 6). Quite in the opposite direction, surgery times were longest in the last two half-year intervals. The complexity of cases in whom robotic assistance was used was stable, with only a slight increase in the proportion of recurrent cases, but also a slight decrease in hiatal defect size. In multivariate analysis, it was analysed which variables predicted failure to reach a textbook outcome. In total, 110 of the 233 patients (47%) did not reach it, but only higher ASA class was associated with a non-textbook outcome (Table 7). Patients with a large hiatal defect and those with recurrent disease also tended to have worse outcomes, but these differences did not reach statistical significance. Finally, the surgical technique (i.e., RALF or CLF) was obviously not linked to reaching a textbook outcome or not.

**Table 6:** The learning curve of robot-assisted fundoplication over time.

	1st half 2023 ^a^ (*n* = 16)	2nd half 2023 (*n* = 19)	1st half 2024 (*n* = 27)	2nd half 2024 (*n* = 19)	1st half 2025 (*n* = 26)	*p* value**
Total duration of surgery (in minutes)	244 ± 56	234 ± 49	225 ± 58	267 ± 54	285 ± 64	0.005
Set up	42 ± 10	42 ± 13	40 ± 13	47 ± 9	46 ± 14	0.28
Procedure	180 ± 53 (range 100 to 315)	170 ± 46 (range 88 to 247)	164 ± 59 (range 71 to 276)	199 ± 52 (range 128 to 320)	217 ± 72 (range 127 to 396)	0.013
Post-procedure	22 ± 10	22 ± 8	20 ± 7	21 ± 7	23 ± 8	0.84
Intraoperative organ lesion or bleeding	1 (6%)	6 (32%)	2 (7%)	3 (16%)	8 (31%)	0.11
Conversions	3 (19%)	0	0	0	3 (12%)	0.029
ICU admission	7 (44%)	8 (42%)	5 (19%)	8 (42%)	7 (27%)	0.26
Length of hospital stay (median, days)	7.5 (IQR 6 to 12.5; range 5 to 28)	7 (IQR 6 to 9; range 5 to 34)	6 (IQR 5 to 7; range 5 to 52)	7 (IQR 6 to 10; range 5 to 46)	6 (IQR 5 to 7.75; range 5 to 26)	0.94
Postop barium enema						
Normal	14 (88%)	15 (79%)	22 (82%)	16 (84%)	18 (69%)	0.62
Stenosis	0	4 (21%)	4 (15%)	2 (11%)	1 (4%)	0.20
Perforation	0	0	0	0	1 (4%)	0.53
Not done	2 (13%)	0	1 (4%)	1 (5%)	6 (23%)	0.054
Postop adverse event						
Dysphagia	0	5 (26%)	3 (11%)	3 (16%)	3 (12%)	0.23
Reflux	3 (19%)	8 (42%)	6 (22%)	6 (32%)	2 (8%)	0.08
Gastroparesis	1 (6%)	3 (16%)	3 (11%)	0	1 (4%)	0.23
Surgical site infection	0	0	1 (4%)	0	1 (4%)	0.72
Incisional hernia	1 (6%)	4 (21%)	1 (4%)	0	0	0.02
Any additional surgery	3 (19%)	2 (11%)	7 (26%)	3 (16%)	4 (15%)	0.72
Readmission						
For recurrency	4 (25%)	4 (21%)	4 (15%)	7 (37%)	2 (8%)	0.16
For hernia repair	1 (6%)	3 (16%)	4 (15%)	1 (5%)	2 (8%)	0.69
Textbook outcome	8 (50%)	8 (42%)	17 (63%)	8 (42%)	16 (62%)	0.45

a Includes first 2 patients operated in late 2022.

**Statistical tests without linear time trend analysis.

**Table 7:** Prediction of no textbook outcome: results of logistic regression (nagelkerke’s r^2^ = 0.09).

Variable	Risk among exposed vs. unexposed	Univariate relative risk (RR) with 95% confidence interval	Multivariate odds ratio (OR) with 95% confidence interval
Robot-assisted surgery	50/107 vs. 60/126	1.0 (0.8 to 1.3)	1.0 (0.6 to 1.9)
Age 60 or older	75/148 vs. 35/85	1.3 (0.9 to 1.8)	1.1 (0.6 to 2.0)
Male sex	31/85 vs. 79/151	0.8 (0.7 to 1.0)^a^	0.6 (0.3 to 1.1)
ASA ≥ 3	43/70 vs. 67/163	1.3 (1.1 to 1.5)^a^	2.2 (1.2 to 3.7)^a^
BMI ≥ 30 kg/m^2^	39/88 vs. 71/145	0.9 (0.8 to 1.1)	1.1 (0.2 to 4.9)
Hiatal defect ≥ 25 cm^2^	43/96 vs. 67/137	0.9 (0.8 to 1.1)	1.3 (0.3 to 5.6)
Recurrency	30/50 vs. 80/183	1.2 (1.0 to 1.3)	1.7 (0.9 to 3.3)

a Statistically significant at the 0.05 level.

## Discussion

Over 20 years ago, it was feared that the clinical effects of robotic fundoplication could change “from theoretic advantages to real problems” (19). In fact, a large registry analysis found a significantly higher complication rate after robot-assisted as compared to laparoscopic hiatal hernia repair (20), but this was partly attributable to learning-curve effects. The present study confirmed that robot-assisted anti-reflux surgery is a safe procedure, if proper training is in place. Nevertheless, RALF was not found to convey any specific advantage. As exemplified by the wide confidence intervals, the present results include the possibility that RALF offers better or worse results than CLF.

Surprisingly, a learning curve was not found in the present study, as operation times and clinical results failed to show any visible improvement over the 5-year study period. Other groups described their learning curve to be as short as 50 cases (21–23). Our operation times are comparable to previous other studies. In a study from Switzerland, for example, median operation time was over 1 h longer (232 vs. 163 min) for robot-assisted as compared to conventional laparoscopic repair of large hiatal hernia (22). Similarly, a US American study found the difference to be 226 vs. 180 min (20), which is very similar to the present findings (251 vs. 177 min). Study results on this outcome are however highly heterogeneous (5), as can be expected from the differences in patient selection and surgical expertise.

Preoperative imaging has a crucial role before anti-reflux surgery. As seen in the present study, computed tomography (CT) is gradually replacing the barium swallow, because CT scanning allows for more accurate evaluation of anatomical structures. The barium swallow does not visualize the diaphragm, which makes this test clearly less useful in patients with suspected hiatal hernia or diaphragmatic deformity after previous surgery. For such reasons, other centres have even moved to three-dimensional CT scanning (24). Barium esophagography, on the other hand, will keep its role as a primary test that helps to understand the association between symptoms and esophageal anatomy (23–26).

This study has several shortcomings. First, the validity of all between-group comparisons is low, because confounding can only partly be controlled for in an observational study. Second, data on other important outcomes were not recorded, such as postoperative pain, health-related quality of life, or costs. With regard to costs, however, it is obvious that the robotic approach significantly increases the costs (8, 27). Accordingly, robot-assisted anti-reflux surgery can only be a cost-effective procedure, when being applied in patients, who can derive real clinical benefits from this technique. In the present study, however, it was not possible to define a subgroup that achieves better outcomes from robot-assistance during surgery.

Valid clinical research comparing RALF with CLF is obviously difficult to perform. Surgeons use robotic assistance mainly for procedures that are technically more demanding. In observational studies of anti-reflux surgery, this leads to selection bias and confounding, because patients with more complex GERD are more likely to undergo RALF. This bias can only partly be corrected for by statistical methods. In the systematic review by Xiao et al., only 1 of the 33 cohort studies was judged to have a low risk of confounding (6). All statistical approaches (multivariate modelling, propensity score matching, or other) are imperfect, because the technical difficulty of an anti-reflux procedure cannot be satisfactorily predicted by preoperative variables. Therefore, we hypothesize that the majority of cohort studies—including our own—suffer from residual confounding which biases results against the robotic approach.

Consequently, it has to be admitted that further RCTs on this topic are required. Only randomization is able to control selection bias and confounding. Proving the benefit of robot-assisted anti-reflux surgery, however, also requires that RCTs are specifically focused on the more complex cases, who are much more likely to benefit from this type of procedure. The existing RCTs do not or only partly address this question. For the Dutch trial, for example, recurrent cases were not eligible (7). All RCTs on this topic were small and conducted more than 20 years ago. In spite of the disappointing RCTs results (7), surgeons continue to trust in the usefulness and superiority of robotic support. We believe that this perseverance of the surgical community is fuelled not by the manufacturers’ marketing, but rather is caused by hands-on experience in the operating room. Future RCTs should be better designed to grasp this concept with a specific focus on selecting more complex cases.

In summary, no specific advantage of robot-assisted anti-reflux surgery could be shown in the present study. Due to the non-randomized study type, however, it remains well possible that such an advantage exists, but was reversed by selection bias. The surgical community should use robotic assistance selectively and focus future studies on such well-selected, complex cases.

## Data Availability

The raw data supporting the conclusions of this article will be made available by the authors, without undue reservation.

## References

[1] Fass R. Gastroesophageal reflux disease. N Engl J Med. (2022) 387:1207–16. 10.1056/NEJMcp2114026 36170502

[2] Maret-Ouda J Markar SR Lagergren J. Gastroesophageal reflux disease: a review. JAMA. (2020) 324:2536–47. 10.1001/jama.2020.21360 33351048

[3] Bais JE Bartelsman JF Bonjer HJ Cuesta MA Go PM Klinkenberg-Knol EC. Laparoscopic or conventional nissen fundoplication for gastro-oesophageal reflux disease: randomised clinical trial. The Netherlands Antireflux Surgery Study Group. Lancet. (2000) 355:170–4. 10.1016/S0140-6736(99)03097-4 10675115

[4] Vertaldi S D'Amore A Manigrasso M Anoldo P Chini A Maione F. Robotic surgery and functional esophageal disorders: a systematic review and meta-analysis. J Pers Med. (2023) 13:231. 10.3390/jpm13020231 36836465 PMC9966072

[5] Gonçalves-Costa D Barbosa JP Quesado R Lopes V Barbosa J. Robotic surgery versus laparoscopic surgery for anti-reflux and hiatal hernia surgery: a short-term outcomes and cost systematic literature review and meta-analysis. Langenbecks Arch Surg. (2024) 409:175. 10.1007/s00423-024-03368-y PMC11156741 38842610

[6] Xiao X Zhang C Li H Chen J Zhang J Yu W. Robotic-assisted versus laparoscopic esophageal hiatal hernia and anti-reflux surgery: a comprehensive systematic review and meta-analysis. Hernia. (2026) 30:273. 10.1007/s10029-026-03770-z 42397587

[7] Draaisma WA Ruurda JP Scheffer RC Simmermacher RK Gooszen HG Jong R- Randomized clinical trial of standard laparoscopic versus robot-assisted laparoscopic nissen fundoplication for gastro-oesophageal reflux disease. Br J Surg. (2006) 93:1351–9. 10.1002/bjs.5535 17058295

[8] Morino M Pellegrino L Giaccone C Garrone C Rebecchi F. Randomized clinical trial of robot-assisted versus laparoscopic nissen fundoplication. Br J Surg. (2006) 93:553–8. 10.1002/bjs.5325 16552744

[9] Müller-Stich BP Reiter MA Mehrabi A Wente MN Fischer L Köninger J. No relevant difference in quality of life and functional outcome at 12 months’ follow-up-a randomised controlled trial comparing robot-assisted versus conventional laparoscopic nissen fundoplication. Langenbecks Arch Surg. (2009) 394:441–6. 10.1007/s00423-008-0446-8 19165497

[10] Lang F Huber A Kowalewski KF Kenngott HG Billmann F Billeter AT. Randomized controlled trial of robotic-assisted versus conventional laparoscopic fundoplication: 12 years follow-up. Surg Endosc. (2022) 36:5627–34. 10.1007/s00464-021-08969-y 35076737 PMC9283162

[11] Nakadi IE Mélot C Closset J DeMoor V Bétroune K Feron P. Evaluation of da vinci nissen fundoplication clinical results and cost minimization. World J Surg. (2006) 30:1050–4. 10.1007/s00268-005-7950-6 16736336

[12] Xia M Zhou M Zhu Z Mao J Wang Z. Robot-assisted versus conventional laparoscopic antireflux surgery: a retrospective cohort analysis of perioperative and medium-term outcomes. J Robot Surg. (2025) 19:348. 10.1007/s11701-025-02526-8 40613965

[13] Benedix F Adolf D Peglow S Gstettenbauer LM Croner R. Short-term outcome after robot-assisted hiatal hernia and anti-reflux surgery-is there a benefit for the patient? Langenbecks Arch Surg. (2021) 406:1387–95. 10.1007/s00423-020-02051-2 33409583

[14] Jiang X Ye C Jiang L Wei G Dai S Xi Y. Single-center experience of transitioning from video-assisted laparoscopic to robotic Heller myotomy with dor fundoplication for esophageal motility disorders. BMC Surg. (2023) 23:341. 10.1186/s12893-023-02202-4 37950218 PMC10638721

[15] Latorre-Rodriguez AR Vittori A Bremner RM Mittal SK. Laparoscopic vs. Robotic-assisted antireflux surgery: a matched cohort analysis of procedure costs and outcomes. Surg Endosc. (2026) 40:2967–75. 10.1007/s00464-025-12551-1 41559337 PMC13053476

[16] Rashid R Sohrabi C Kerwan A Franchi T Mathew G Nicola M. The STROCSS 2024 guideline: strengthening the reporting of cohort, cross-sectional, and case-control studies in surgery. Int J Surg. (2024) 110:3151–65. 10.1097/JS9.0000000000001268 38445501 PMC11175759

[17] Köckerling F Zarras K Adolf D Kraft B Jacob D Weyhe D. What is the reality of hiatal hernia management?-A registry analysis. Front Surg. (2020) 7:584196. 10.3389/fsurg.2020.584196 PMC7642514 33195390

[18] Analatos A Håkanson BS Ansorge C Lindblad M Lundell L Thorell A. Hiatal hernia repair with tension-free mesh or crural sutures alone in antireflux surgery: a 13-year follow-up of a randomized clinical trial. JAMA Surg. (2024) 159:11–8. 10.1001/jamasurg.2023.4976 37819652 PMC10568445

[19] Costi R Himpens J Bruyns J Cadière GB. Robotic fundoplication: from theoretic advantages to real problems. J Am Coll Surg. (2003) 197:500–7. 10.1016/S1072-7515(03)00479-4 12946806

[20] Klock JA Walters RW Nandipati KC. Robotic hiatal hernia repair associated with higher morbidity and readmission rates compared to laparoscopic repair: 10-year analysis from the national readmissions database (NRD). J Gastrointest Surg. (2023) 27:489–97. 10.1007/s11605-022-05548-x 36508133

[21] Wilhelm A Nocera F Schneider R Koechlin L Daume DL Fourie L. Robot-assisted vs. Laparoscopic repair of complete upside-down stomach hiatal hernia (the RATHER-study): a prospective comparative single center study. Surg Endosc. (2022) 36:480–8. 10.1007/s00464-021-08307-2 33523279

[22] Elmously A Gray KD Ullmann TM Fahey TJ 3rd Afaneh C Zarnegar R. Robotic reoperative anti-reflux surgery: low perioperative morbidity and high symptom resolution. World J Surg. (2018) 42:4014–21 10.1007/s00268-018-4708-5 29959490

[23] Menke V Kottmann T Willeke F Hansen O. Learning curves and procedural times in senhance®-robotic assisted fundoplication: results from 237 consecutive patients undergoing robotic fundoplication in a single center as part of the European TRUST robotic surgery registry study. Surg Endosc. (2023) 37:8254–62. 10.1007/s00464-023-10226-3 37670190

[24] Santana AV Herbella FAM Domene CE Volpe P Neto W Polízio RP. High-resolution 3-dimensional tomography may be a useful tool for understanding the anatomy of hiatal hernias and surgical planning of patients eligible for laparoscopic or robotic antireflux surgery. Surg Endosc. (2024) 38:780–6. 10.1007/s00464-023-10599-5 38057539

[25] Levine MS Carucci LR DiSantis DJ Einstein DM Hawn MT Martin-Harris B. Consensus statement of society of abdominal radiology disease-focused panel on barium esophagography in gastroesophageal reflux disease. AJR Am J Roentgenol. (2016) 207:1009–15. 10.2214/AJR.16.16323 27490234

[26] Bauerle W Franey D Allsbrook A Evans J Reese V Stoltzfus J. Retrospective cost analysis of robotic and laparoscopic anti-reflux surgery and paraesophageal hernia repair. Surg Endosc. (2024) 38:7179–86. 10.1007/s00464-024-11294-9 39367133

[27] Ross SB Sucandy I Trotto M Christodoulou M Pattilachan TM Jattan J. A decade of experience with minimally invasive anti-reflux operations: robot vs. LESS. Surg Endosc. (2024) 38:2641–8. 10.1007/s00464-024-10771-5 38503903

